# Central Nervous System Tuberculoma in Qatar: Clinical, Radiological, and Pathological Insights From a Rare Case With Literature Review

**DOI:** 10.7759/cureus.113780

**Published:** 2026-08-01

**Authors:** Mohammad Mohsin Arshad, Malavika Kumar, Devika Kumar, Muhammad Junaid Iqbal, Sarah Sayed, Khair Muhammad

**Affiliations:** 1 Radiology, Hamad General Hospital, Doha, QAT; 2 College of Medicine, Weill Cornell Medicine Qatar, Doha, QAT; 3 Emergency, Hamad General Hospital, Doha, QAT; 4 Pathology, Hamad Medical Corporation, Doha, QAT

**Keywords:** anti-tuberculosis therapy, central nervous system tuberculosis, intracranial tuberculoma, magnetic resonance imaging, ring-enhancing lesion

## Abstract

Intracranial tuberculomas are an exceptionally rare manifestation of tuberculosis in Qatar and the Gulf Cooperation Council (GCC) region; their presentation can closely mimic other intracranial pathologies, posing diagnostic challenges. A 24-year-old Indian woman presented with headache and new-onset seizures, with a prior history of mediastinal lymphadenopathy. Neuroimaging revealed multiple intracranial ring-enhancing lesions associated with surrounding edema and midline shift. Based on the clinical and radiological findings, anti-tuberculosis therapy was initiated promptly. Serial follow-up imaging demonstrated significant regression and eventual resolution of the lesions, correlating with clinical improvement. Intracranial tuberculomas remain rare in the GCC, often presenting with nonspecific neurological symptoms and atypical ring-enhancing brain lesions. Clinicians must maintain a high degree of suspicion, as early recognition and timely initiation of therapy are crucial to achieve complete lesion resolution and avoid invasive interventions.

## Introduction

Central nervous system (CNS) tuberculomas represent an uncommon manifestation of extrapulmonary tuberculosis (TB) [1]. Although tuberculosis incidence is low in the Gulf Cooperation Council (GCC) region, including Qatar, increasing population mobility from TB-endemic countries presents a diagnostic challenge, as CNS tuberculoma may occur in individuals with prior exposure despite residing in a low-incidence setting. Their clinical and radiological presentation is highly variable and frequently mimics other intracranial pathologies such as metastases, primary brain tumors, abscesses, or neurocysticercosis, often leading to diagnostic delay [2]. Neuroimaging, particularly magnetic resonance imaging (MRI), plays a pivotal role in the early detection, characterization, and longitudinal assessment of these lesions, while also helping differentiate them from other intracranial pathologies and monitor response to therapy [2].

Despite the importance of early recognition, only a limited number of intracranial tuberculoma cases have been reported from Qatar and the wider GCC region, leaving regional experience with their diagnosis and management scarce. This case adds to the limited literature by illustrating the clinicopathological correlation, characteristic imaging evolution, and favorable response to early anti-tuberculosis therapy in a young patient with microbiologically confirmed tuberculosis. By integrating the clinical course with radiological, pathological, and microbiological findings, this report highlights important diagnostic considerations and reinforces the need to include CNS tuberculoma in the differential diagnosis of ring-enhancing intracranial lesions in patients with epidemiological risk factors or with evidence of systemic tuberculosis.

## Case presentation

A 24-year-old woman who had lived in India before migrating to Qatar 2.5 years earlier presented to the emergency department with a 10-day history of mild headache followed by a single generalized tonic-clonic seizure. She was alert and oriented, with no focal neurological deficits.

**Figure 1:**
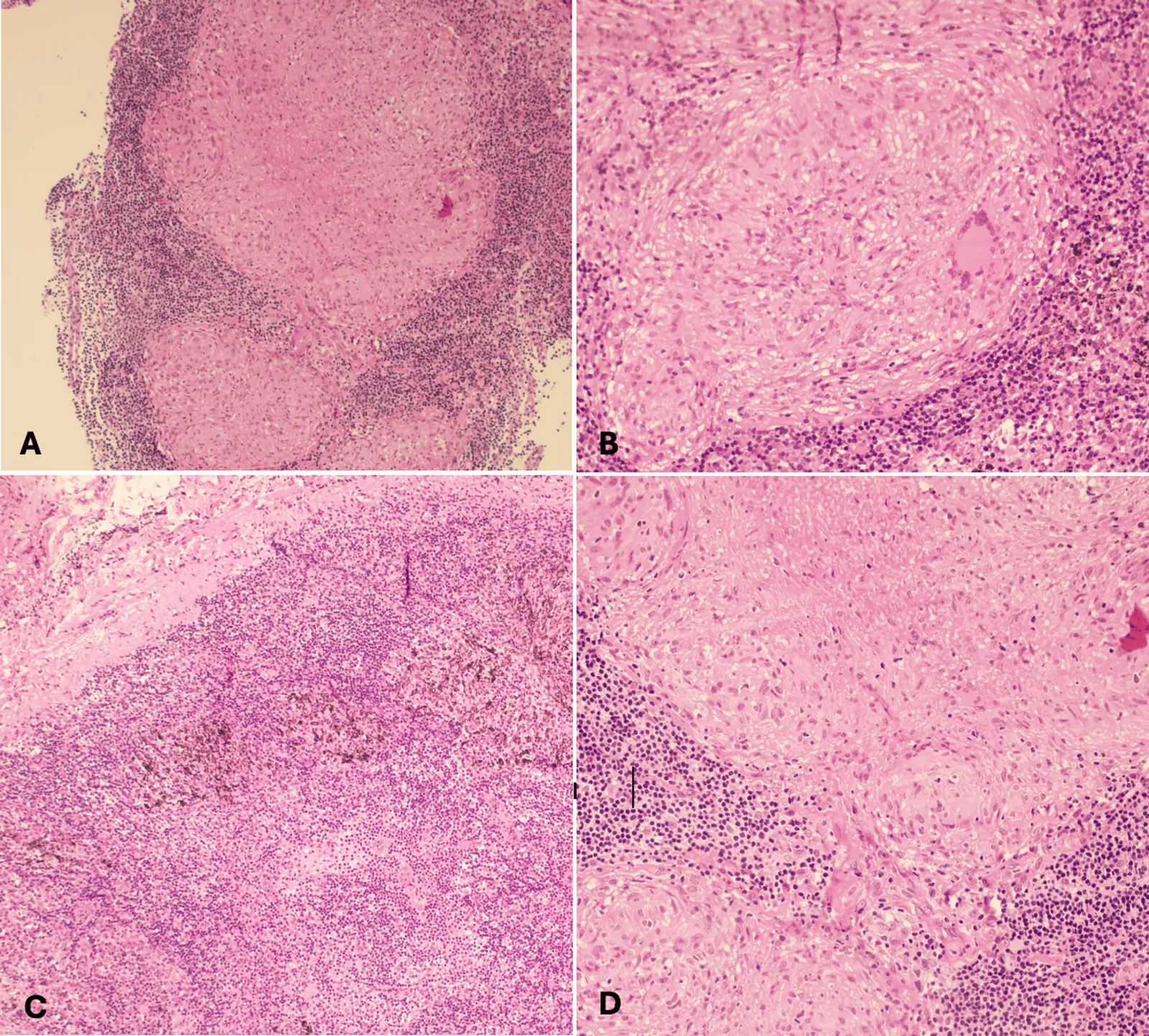
Histopathological features of necrotizing granulomatous lymphadenitis. (A) Low-power view demonstrating multiple well-formed granulomas partially replacing the lymph node parenchyma (H&E, ×40). (B) Higher magnification showing granulomas composed of epithelioid histiocytes and multinucleated giant cells surrounding central necrotic debris (H&E, ×100). (C) and (D) Representative sections demonstrating confluent granulomatous inflammation with peripheral lymphocytic cuffing and central necrosis (H&E, ×100–200).

Several months before presentation, she was investigated for an unprovoked right internal jugular vein thrombosis. Contrast-enhanced CT of the neck demonstrated necrotic paratracheal and superior mediastinal lymphadenopathy. Lymph node biopsy revealed necrotizing granulomatous lymphadenitis with well-formed granulomas, caseous necrosis, epithelioid histiocytes, and multinucleated giant cells (Figures 1A-1D). Tuberculosis polymerase chain reaction (PCR) was positive, acid-fast bacilli smear was negative, and drug susceptibility testing confirmed sensitivity to first-line anti-tuberculosis agents except pyrazinamide.

**Figure 2:**
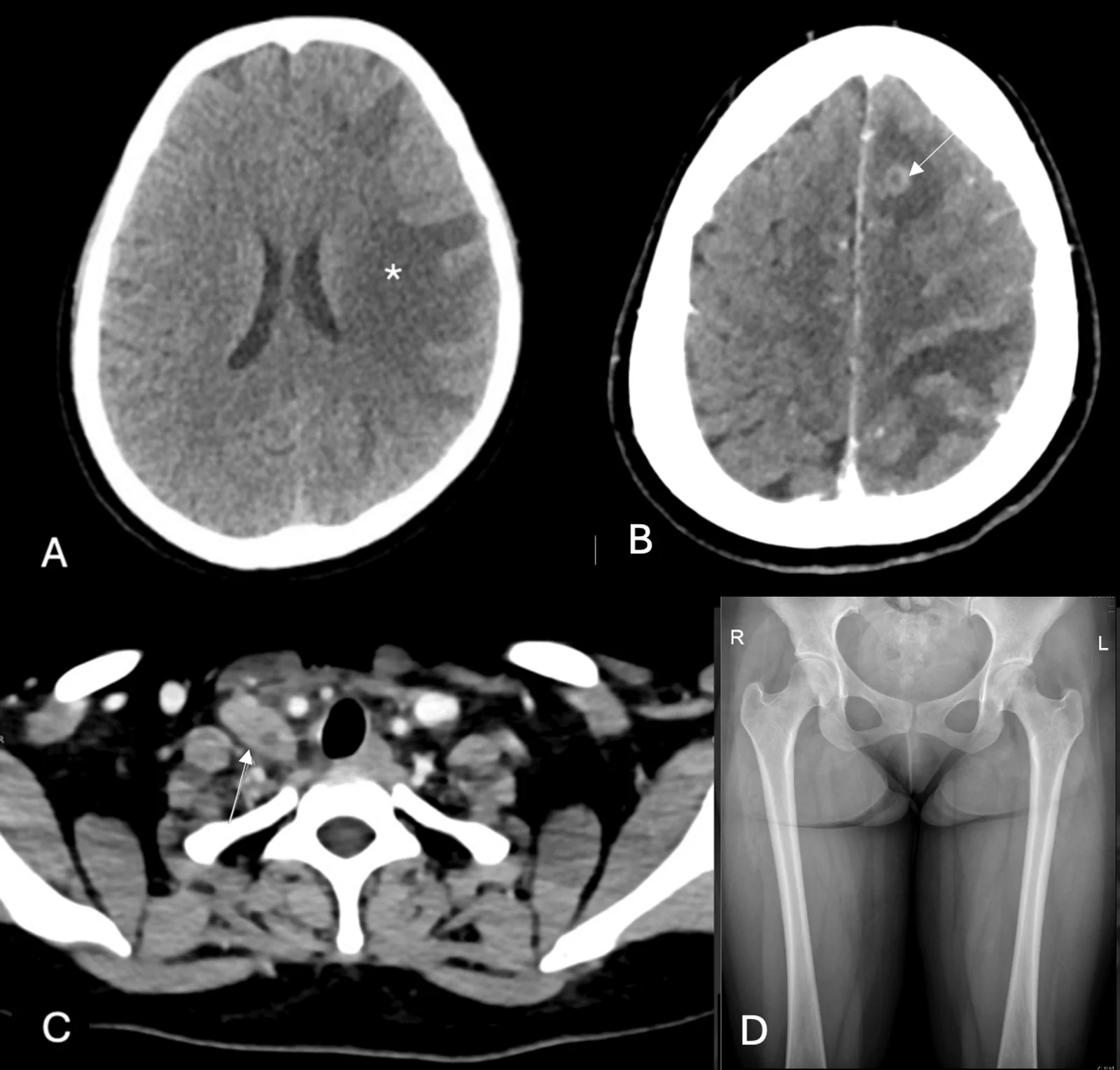
Initial imaging findings. (A) Non-contrast CT head demonstrates a large area of hypoattenuation (asterisk) in the left frontoparietotemporal region. (B) Contrast-enhanced CT of the brain shows multiple well-defined ring-enhancing lesions (arrow) in the left cerebral hemisphere with surrounding vasogenic edema and sulcal effacement. (C) Contrast-enhanced CT of the neck demonstrates necrotic right superior mediastinal (paratracheal) lymphadenopathy (arrow). (D) Skeletal survey of the extremities demonstrates no evidence of calcified intramuscular cysticerci suggestive of neurocysticercosis.

Following her seizure, non-contrast CT of the brain demonstrated a hypodense lesion in the left frontoparietotemporal region with surrounding edema. Subsequent contrast-enhanced CT revealed multiple ring-enhancing lesions involving the left cerebral hemisphere and right paramedian pons, along with persistent necrotic mediastinal lymphadenopathy. Skeletal imaging showed no evidence of neurocysticercosis (Figures 2A-2D).

**Figure 3:**
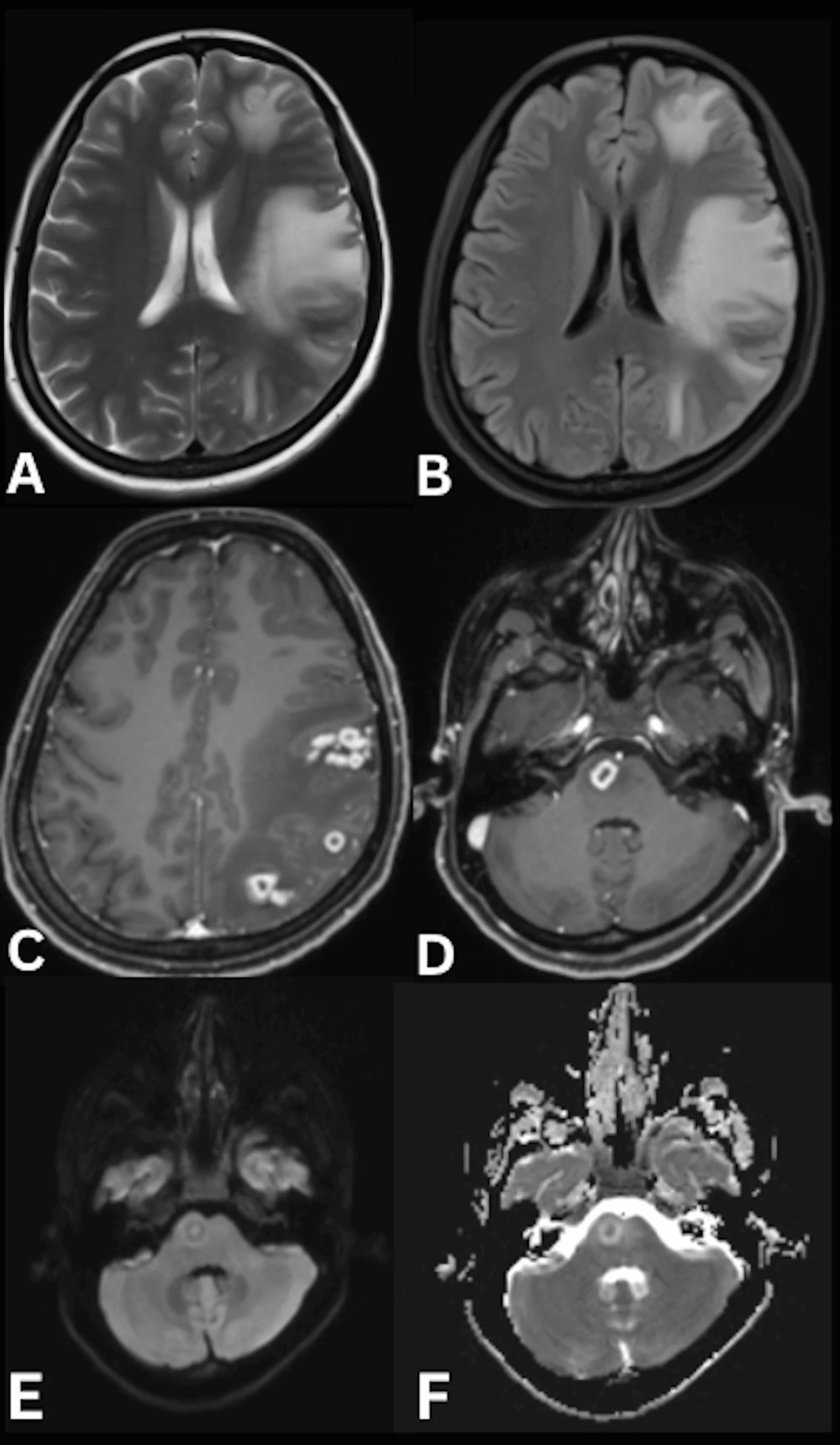
Pre-treatment contrast-enhanced MRI of the brain. (A) Axial T2-weighted, (B) axial FLAIR, (C) and (D) axial post-contrast T1-weighted, and (E) and (F) diffusion-weighted imaging (DWI) and apparent diffusion coefficient (ADC) sequences demonstrate multiple clustered ring-enhancing lesions in the left frontoparietal region, predominantly involving the subcortical white matter, along with a separate enhancing lesion in the right pons. Smaller lesions demonstrate nodular enhancement, whereas the larger lesions demonstrate central diffusion restriction. Moderate surrounding vasogenic edema on the FLAIR images. FLAIR: fluid-attenuated inversion recovery.

Contrast-enhanced MRI of the brain confirmed multiple ring-enhancing lesions predominantly involving the left frontoparietal subcortical white matter and right paramedian pons, with associated vasogenic edema and mild mass effect (Figures 3A-3D). The main radiological differentials included cerebral abscesses, metastatic disease, high-grade glioma, and neurocysticercosis. However, in the setting of microbiologically confirmed systemic tuberculosis, the imaging findings were highly suggestive of intracranial tuberculomas.

A diagnosis of CNS tuberculomas was established based on the clinicoradiological and microbiological findings. The patient received a modified anti-tuberculosis regimen comprising rifampicin, isoniazid, moxifloxacin, and pyridoxine for 12 months due to pyrazinamide resistance. Adjunctive dexamethasone was administered with gradual tapering, and levetiracetam was continued for seizure control. Treatment adherence was monitored through a video-directed observed therapy (vDOT) program.

**Figure 4:**
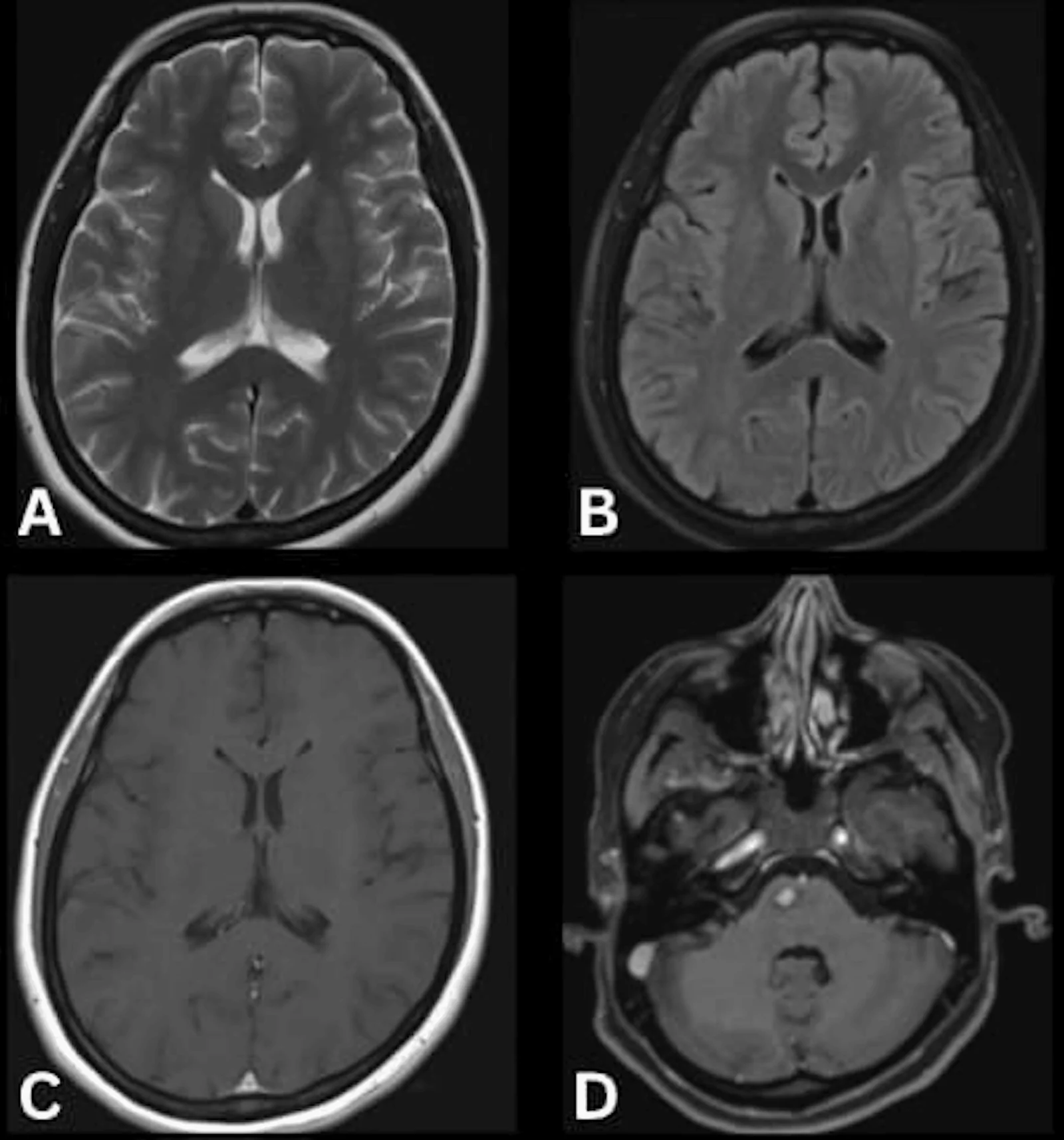
Post-treatment contrast-enhanced MRI of the brain. (A) Axial T2-weighted, (B) axial FLAIR, and (C) and (D) axial post-contrast T1-weighted images demonstrate a marked reduction in the number and size of the bilateral cerebral enhancing lesions, with significantly decreased surrounding vasogenic edema. The enhancing lesion in the right pons has also markedly decreased in size. No ventriculomegaly or midline shift is identified. FLAIR: fluid-attenuated inversion recovery.

Follow-up contrast-enhanced brain MRI demonstrated marked regression of the intracranial lesions with resolution of vasogenic edema and mass effect, consistent with a favorable response to therapy (Figures 4A-4D).

## Discussion

Central nervous system (CNS) tuberculosis caused by *Mycobacterium tuberculosis* is an uncommon but potentially devastating manifestation of tuberculosis, accounting for up to 10% of all tuberculosis cases and associated with significant morbidity and mortality [1]. Intracranial tuberculomas are well-circumscribed, avascular granulomatous lesions that may occur as solitary or multiple intracranial masses [1]. Their distribution varies with age, with infratentorial involvement more frequently observed in pediatric patients and supratentorial lesions predominating in adults. In adults, lesions commonly occur at the gray-white matter junction, likely due to hematogenous dissemination through small penetrating vessels and subsequent granuloma formation [2]. Histopathologically, tuberculomas consist of epithelioid granulomas with central caseous necrosis surrounded by inflammatory cells and may contain *Mycobacterium tuberculosis* organisms [3]. Although uncommon in non-endemic regions, intracranial tuberculomas have been reported in approximately one in 300 untreated pulmonary tuberculosis cases and may account for a substantial proportion of intracranial mass lesions in endemic settings [4].

In the present case, a young adult woman with a history of necrotizing granulomatous mediastinal lymphadenitis presented with headache and new-onset seizure after residing in Qatar for 2.5 years following migration from India. The combination of epidemiological background, microbiologically confirmed extracranial tuberculosis, and characteristic intracranial lesions facilitated early diagnosis. CNS tuberculoma remains exceptionally rare in Qatar and the wider Gulf Cooperation Council (GCC) region, with limited adult cases reported in the literature. Previously reported cases demonstrated variability in demographic characteristics, presenting symptoms, imaging findings, and therapeutic approaches. The clinical course of our patient, from presentation with headache and seizure to diagnostic confirmation, initiation of anti-tuberculosis therapy, and subsequent radiological resolution, highlights the importance of early recognition and timely management.

Clinically, intracranial tuberculomas may remain asymptomatic until lesion enlargement results in mass effect or increased intracranial pressure. Reported neurological manifestations include headache, seizures, vomiting, focal neurological deficits, and papilledema [3]. In our patient, seizure was the presenting neurological manifestation, reflecting cortical involvement by multiple supratentorial lesions.

The diagnosis of intracranial tuberculoma remains challenging because microbiological confirmation from the central nervous system is frequently difficult to obtain. Cerebrospinal fluid (CSF) analysis may be normal in isolated parenchymal disease, and conventional investigations such as Ziehl-Neelsen staining and culture often demonstrate limited sensitivity. CSF PCR may improve diagnostic yield but can have delayed availability and variable sensitivity [3,4]. Therefore, diagnosis frequently relies on integration of clinical, epidemiological, microbiological, pathological, and radiological findings. In this case, prior lymph node biopsy confirming tuberculosis provided critical diagnostic evidence, allowing treatment initiation without invasive neurosurgical biopsy.

Neuroimaging plays a central role in the diagnosis and follow-up of intracranial tuberculomas. On CT, lesions may appear as iso- or hypodense masses with variable enhancement patterns and surrounding vasogenic edema [4]. MRI provides improved tissue characterization and demonstrates the spectrum of tuberculoma evolution based on the degree of caseation and liquefaction. Typical findings include ring enhancement, a T2-hypointense rim related to caseation, and variable diffusion characteristics depending on lesion composition [2,3]. In our patient, the combination of multiple ring-enhancing lesions, surrounding edema, and associated systemic tuberculosis favored the diagnosis of intracranial tuberculomas.

Due to their nonspecific imaging appearance, intracranial tuberculomas frequently mimic other ring-enhancing intracranial lesions, including cerebral abscesses, metastases, high-grade gliomas, neurocysticercosis, toxoplasmosis, and inflammatory conditions [2]. Differentiation may be aided by advanced MRI techniques when diagnostic uncertainty persists. Magnetic resonance spectroscopy may demonstrate lipid-lactate peaks with relatively mild choline elevation in tuberculomas, whereas neoplastic lesions typically demonstrate increased choline metabolites [5]. Similarly, reduced perfusion parameters combined with characteristic spectroscopy findings may help distinguish tuberculomas from high-grade gliomas and metastases [6].

The standard treatment of CNS tuberculosis consists of prolonged combination anti-tuberculosis therapy with adjunctive corticosteroids to reduce the inflammatory response and intracranial complications [1,3]. In this case, a modified regimen consisting of rifampicin, isoniazid, moxifloxacin, and pyridoxine was selected because susceptibility testing demonstrated pyrazinamide resistance. The inclusion of moxifloxacin provided additional bactericidal activity and allowed continuation of an effective multidrug regimen despite pyrazinamide exclusion. Corticosteroid therapy was administered because of significant surrounding edema and mass effect. Serial MRI demonstrated marked regression of lesions and resolution of associated edema, supporting the effectiveness of medical therapy.

Follow-up imaging remains essential in monitoring treatment response, identifying paradoxical reactions, and guiding treatment duration [7]. Although most tuberculomas respond favorably to medical therapy, larger lesions, multiple lesions, or lesions with significant mass effect may require prolonged treatment and close clinical-radiological surveillance. Surgical intervention remains reserved for cases with diagnostic uncertainty, progressive neurological deterioration, or complications requiring decompression. A summary of reported adult cases from Qatar and other GCC countries is provided in Table 1.

**Table 1:** Reported adult cases of central nervous system tuberculoma in Qatar and other GCC region. LP: lumbar puncture; SWI: susceptibility-weighted imaging; GCC: Gulf Cooperation Council; RIF: rifampicin, INH: isoniazid.

Study	Year	Region	Age	Symptoms/signs	Imaging finding	How diagnosed	Management	Outcome
Ahmed et al. [8]	2023	Qatar	41	Spasm of left arm and leg followed by weakness and numbness -Headache and nausea	CT Head: Ill-defined hyperdense lesion in the right high parafalcine region. MRI Head: Lobulated extra-axial dural-based lesion	Intraoperative brain biopsy: necrotizing granulomatous inflammation; TB QuantiFERON: positive; TB culture: *Mycobacterium tuberculosis* complex	Standard anti-TB four-drug regimen	No follow-up
Safi et al. [9]	2019	Qatar	38	Worsening headaches and blurring of vision	CT Head: Left frontal and basal ganglia isodense lesions with small hypodense areas, associated perilesional edema, mass effect, and 10 mm midline shift. MRI Head: Multiple left frontal and basal ganglia lesions; T2/FLAIR iso- to hypointense, T1 central isointense with peripheral hyperintensity; post-contrast images show thick nodular peripheral enhancement; no central diffusion restriction; small SWI blooming foci	Intraoperative frozen section: granulomatous lesion with caseous necrosis, suggestive of tuberculoma. Tissue culture: positive for *Mycobacterium tuberculosis* complex polymerase chain reaction (PCR): positive for tuberculosis	Left pterional craniotomy and complete surgical resection of the left frontal lesion. Antituberculous medications with four-drug regimen for one year	Serial imaging showed no residual/recurrence of disease at one-year follow-up
Sadek et al. [10]	2024	Qatar	37	Unilateral vision loss that subsequently progressed to bilateral vision loss, with left-sided headache. Later loss of consciousness, generalized tonic-clonic seizure, right-sided weakness and aphasia	CT Head: No evidence of acute intracranial abnormalities. MRI head: leptomeningitis with intra-axial tuberculomas	LP showed elevated white blood cells with lymphocytic predominance.	Standard anti-TB four-drug regimen plus steroid	Follow-up brain MRI demonstrated significant regression of leptomeningeal inflammation with complete resolution of the tuberculomas
Alharbi et al. [11]	2021	Saudi Arabia	67	Nausea and vomiting, imbalance, dizziness, weight loss, and night sweats.	CT Head: Diffuse right parietal, occipital, and cerebellar white matter hypodensity with significant surrounding edema. MRI Head: Well-defined lesions in the medial right parietal lobe and right cerebellar hemisphere; T2/FLAIR shows hyperintense rim with hypointense center. Chest CT: Bilateral pulmonary nodules with prominent reactive thoracic lymph nodes. Abdomen/Pelvis CT: Multiple enlarged abdominal lymph nodes	Brain biopsy (cerebellar lesion): caseous necrotic center with surrounding lymphocytes, epithelioid cells, and multinucleated Langhans-type giant cells tuberculosis PCR: positive acid-fast bacilli (AFB) culture: positive for *Mycobacterium tuberculosis*	Sub-occipital craniotomy for biopsy and lesion resection. Anti-tuberculosis therapy.	On six-month follow-up, he was independent, ambulatory, with mild cerebellar dysfunction
Atteiah et al. [12]	2024	Saudi Arabia	45	Generalized tonic-clonic convulsion	CT Brain: Hypodense lesion in the right frontal lobe involving gray and white matter, with no post-contrast enhancement. MRI Brain: Ill-defined, enhancing meningeal-based lesion	Histopathology: diffuse granulomatous inflammation with focal caseous necrosis, consistent with tuberculosis	Right frontal craniotomy with complete excision of the mass and attached dura. Medical therapy: anti-tuberculosis medications and phenytoin for seizure control	Not reported
Sharma et al. [13]	2012	Oman	25	Double vision on right lateral gaze	MRI Brain: Ring-enhancing lesion in the ventral midbrain at the level of the superior colliculus (tegmentum) with surrounding perilesional edema	None	Medical therapy with a four-drug anti-tuberculosis regimen for one year	Follow-up MRI after one and two years showed complete resolution of the lesion
Sharma et al. [13]	2012	Oman	48	Inability to turn his eyes toward the left side	MRI Brain: Intensely enhancing nodular lesions, approximately 1 cm in diameter, located in the pons	Image-guided biopsy of the lesion showed caseating granuloma, which on special staining showed acid-fast bacilli	Medical therapy with a four-drug anti-tuberculosis regimen	Follow-up MRI at six months showed disappearance of the lesion
Our case	2026	Qatar	24	Headache and new-onset seizures, with a prior history of mediastinal lymphadenopathy	CT Brain: Large hypodense area in the left frontoparietotemporal white matter causing mass effect with 3.2 mm rightward midline shift. MRI Brain: Multiple small ring-enhancing lesions in the left frontoparietal subcortical region; central T2 hyperintensity with peripheral hypointense rims on T1/T2, wall thickening, susceptibility blooming, and central diffusion restriction in larger lesions	Lymph node biopsy revealed necrotizing granulomatous lymphadenitis with positive tuberculosis polymerase chain reaction (PCR) and negative acid-fast bacilli smear	Modified anti-TB regimen (RIF/INH/moxifloxacin/pyridoxine) for one year	Follow-up MRI marked reduction in the number and size of intracranial lesions, resolution of vasogenic edema

## Conclusions

This case highlights a rare presentation of central nervous system (CNS) tuberculoma and emphasizes the importance of considering tuberculosis in the differential diagnosis of ring-enhancing intracranial lesions, particularly in patients with systemic disease or relevant epidemiological risk factors. The nonspecific clinical presentation and overlapping imaging features with neoplastic and infectious pathologies can make diagnosis challenging. Neuroimaging plays a central role in diagnosis and follow-up. Contrast-enhanced MRI with diffusion and susceptibility-weighted imaging provides valuable diagnostic information, while serial imaging allows assessment of treatment response and disease progression. Early integration of clinical, microbiological, and radiological findings enables timely initiation of anti-tuberculosis therapy and favorable outcomes. This case underscores the need for heightened clinical awareness and early diagnostic consideration of CNS tuberculoma, as prompt recognition and treatment can lead to favorable outcomes in this potentially reversible but life-threatening disease.
